# Supplementary material for: Durability of mRNA-1273 against COVID-19 in the time of Delta: Interim results from an observational cohort study
Source: PLoS One. 2022 Apr 28;17(4):e0267824. doi: 10.1371/journal.pone.0267824 (PMC9049574; doi:10.1371/journal.pone.0267824)
Supplement: S1 Table — (PDF) [file pone.0267824.s002.pdf]

Supplementary Information

Table of Contents

Supplementary Table 1. Incidence rate, hazard ratio, and vaccine effectiveness of 2 doses of mRNA-1273 vaccine in preventing SARS-CoV-2 infection and COVID-19 hospitalization, stratified by age, by months after vaccination 2

S1 Table. Incidence rate, hazard ratio, and vaccine effectiveness of 2 doses of mRNA-1273 vaccine in preventing SARS-CoV-2 infection and COVID-19 hospitalization, stratified by age, by months after vaccination

	Vaccinated			Unvaccinated			Hazard Ratio (95% CI)		VE % (95% CI)	
**Outcomes**	**N**	**Number of cases**	**Incidence per 1000 person-years (95% CI)**	**N**	**Number of cases**	**Incidence per 1000 person-years (95% CI)**	**Unadjusted**	**Adjusted[a]**	**Unadjusted**	**Adjusted[a]**
SARS-CoV-2 infection										
0-<2 months	927004	528	4.36 (4.00-4.75)	927004	2786	36.14 (34.82-37.51)	0.12 (0.11-0.13)	0.12 (0.11-0.13)	88.4 (87.3-89.4)	88.0 (86.8-89.1)
2-<4 months	916323	2556	17.02 (16.37-17.69)	469386	9813	141.55 (138.78-144.38)	0.12 (0.11-0.12)	0.16 (0.15-0.16)	88.2 (87.6-88.7)	84.5 (83.8-85.2)
4-<6 months	843804	3455	33.46 (32.36-34.60)	336656	4022	132.60 (128.57-136.77)	0.25 (0.24-0.26)	0.23 (0.22-0.24)	75.1 (73.9-76.2)	77.0 (75.7-78.2)
6-<8 months	326241	1141	41.11 (38.80-43.57)	40040	188	92.30 (80.01-106.49)	0.46 (0.39-0.54)	0.25 (0.20-0.30)	53.8 (46.0-60.5)	75.5 (70.4-79.7)
Aged 18-64 years old										
0-<2 months	689202	418	4.65 (4.22-5.11)	689202	2373	39.86 (38.29-41.50)	0.11 (0.10-0.12)	0.12 (0.10-0.13)	88.9 (87.7-90.0)	88.4 (87.1-89.6)
2-<4 months	680159	2293	20.65 (19.82-21.51)	361409	8968	169.98 (166.50-173.53)	0.12 (0.11-0.13)	0.15 (0.15-0.16)	88.1 (87.5-88.6)	84.6 (83.9-85.4)
4-<6 months	613565	2481	36.36 (34.96-37.82)	247334	2694	145.35 (139.96-150.94)	0.24 (0.23-0.26)	0.22 (0.21-0.24)	75.9 (74.5-77.2)	77.6 (76.0-79.0)
6-<8 months	164711	811	50.69 (47.32-54.30)	2386	22	152.32 (100.29-231.33)	0.33 (0.22-0.51)	0.28 (0.18-0.45)	66.6 (49.0-78.2)	71.6 (54.7-82.2)
Aged ≥65 years old										
0-<2 months	237802	110	3.53 (2.93-4.26)	237802	413	23.53 (21.36-25.91)	0.15 (0.12-0.19)	0.13 (0.11-0.17)	84.7 (81.1-87.6)	86.5 (83.2-89.2)
2-<4 months	236164	263	6.71 (5.95-7.58)	107977	845	51.01 (47.68-54.57)	0.13 (0.11-0.15)	0.16 (0.14-0.18)	87.1 (85.2-88.8)	84.2 (81.7-86.3)
4-<6 months	230239	974	27.81 (26.12-29.61)	89322	1328	112.58 (106.69-118.80)	0.24 (0.22-0.27)	0.23 (0.21-0.26)	75.6 (73.5-77.5)	76.8 (74.5-78.8)
6-<8 months	161530	330	28.08 (25.21-31.28)	37654	166	87.72 (75.34-102.14)	0.33 (0.27-0.39)	0.22 (0.18-0.28)	67.4 (60.5-73.0)	77.7 (72.3-82.1)
COVID-19 hospitalization										
0-<2 months	927004	21	0.17 (0.11-0.27)	927004	301	3.90 (3.48-4.36)	0.04 (0.03-0.07)	0.04 (0.03-0.06)	95.8 (93.5-97.3)	95.9 (93.5-97.4)
2-<4 months	916826	55	0.37 (0.28-0.48)	471487	1226	17.46 (16.51-18.46)	0.02 (0.02-0.03)	0.03 (0.02-0.03)	97.9 (97.3-98.4)	97.4 (96.6-98.0)
4-<6 months	846468	132	1.27 (1.07-1.51)	344860	771	24.77 (23.08-26.58)	0.05 (0.04-0.06)	0.05 (0.04-0.06)	95.1 (94.1-95.9)	94.8 (93.6-95.7)
6-<8 months	328421	36	1.29 (0.93-1.78)	40715	63	30.41 (23.76-38.93)	0.05 (0.03-0.07)	0.06 (0.03-0.09)	95.3 (92.8-96.9)	94.5 (90.9-96.7)
Aged 18-64 years old										

0-<2 months	689202	11	0.12 (0.07-0.22)	689202	195	3.27 (2.84-3.76)	0.03 (0.02-0.06)	0.04 (0.02-0.07)	96.5 (93.6-98.1)	96.2 (93.0-98.0)
2-<4 months	680563	30	0.27 (0.19-0.39)	363280	957	17.86 (16.76-19.03)	0.01 (0.01-0.02)	0.02 (0.01-0.03)	98.5 (97.9-99.0)	98.1 (97.3-98.7)
4-<6 months	615914	35	0.51 (0.37-0.71)	254857	306	15.95 (14.26-17.84)	0.03 (0.02-0.05)	0.03 (0.02-0.05)	96.5 (95.0-97.6)	96.6 (95.0-97.8)
6-<8 months	166067	8	0.49 (0.25-0.99)	2460	2	13.48 (3.37-53.91)	0.04 (0.01-0.18)	0.01 (0.00-0.21)	96.2 (82.0-99.2)	98.7 (78.6-99.9)
Aged ≥65 years old										
0-<2 months	237802	10	0.32 (0.17-0.60)	237802	106	6.03 (4.99-7.30)	0.05 (0.03-0.10)	0.05 (0.02-0.09)	94.8 (90.0-97.3)	95.2 (90.6-97.5)
2-<4 months	236263	25	0.64 (0.43-0.94)	108207	269	16.17 (14.35-18.23)	0.04 (0.03-0.06)	0.05 (0.03-0.08)	96.1 (94.2-97.4)	95.0 (92.4-96.7)
4-<6 months	230554	97	2.76 (2.26-3.37)	90003	465	38.96 (35.58-42.67)	0.07 (0.06-0.09)	0.07 (0.05-0.09)	93.0 (91.2-94.3)	93.2 (91.4-94.6)
6-<8 months	162354	28	2.37 (1.63-3.43)	38255	61	31.71 (24.68-40.76)	0.08 (0.05-0.12)	0.06 (0.04-0.10)	92.4 (88.0-95.2)	93.8 (89.8-96.3)

[a] Adjusted for covariates age, sex, race/ethnicity, index date (in months), number of outpatient and virtual visits, preventive care, KPSC physician/employee status, medical center area.
N = sample size; CI = confidence interval; VE = vaccine effectiveness
